# Impact of *VKORC1*, *CYP4F2* and *NQO1* gene variants on warfarin dose requirement in Han Chinese patients with catheter ablation for atrial fibrillation

**DOI:** 10.1186/s12872-018-0837-x

**Published:** 2018-05-18

**Authors:** Jiao Li, Wenlong Yang, Zhonghui Xie, Kun Yu, Yuhua Chen, Kaijun Cui

**Affiliations:** 1 College of Life Science, Sichuan Normal University, Chengdu, People’s Republic of China; 2 Department of Cardiology, West China Hospital, Sichuan University, Chengdu, People’s Republic of China; 3 State Key Laboratory of Biotherapy/Collaborative Innovation Center for Biotherapy, West China Hospital, Sichuan University, Chengdu, People’s Republic of China; 4 Department of Cardiac Surgery, West China Hospital of Sichuan University, Chengdu, China

**Keywords:** VKORC1, CYP4F2, NQO1, Catheter ablation, Atrial fibrillation, Warfarin

## Abstract

**Background:**

The anticoagulation of atrial fibrillation catheter ablation during the perioperative stage does matter and should be treated with discretion. We aimed to assess impact of three important genes participating in vitamin K cycle (i.e. *VKORC1* rs9923231, *CYP4F2* rs2108622 and *NQO1* rs1800566) on the daily stable warfarin dose requirement in Sichuan Han Chinese patients with catheter ablation of atrial fibrillation.

**Methods:**

A total of 222 atrial fibrillation patients taking stable warfarin therapy after catheter ablation operation were enrolled in this study. The study population included had high (≥2) risk according to the CHA2DS2-VASc risk score. Genotypes of *VKORC1* rs9923231, *CYP4F2* rs2108622 and *NQO1* rs1800566 were analyzed by using the polymerase chain reaction restriction fragment length polymorphism method (PCR-RFLP). Multiple linear regression analysis was applied to depict the impact of *VKORC1* rs9923231, *CYP4F2* rs2108622 and *NQO1* rs1800566 on the daily stable warfarin dose requirement.

**Results:**

Carriers of *VKORC1* rs9923231 AG/GG genotypes required significantly higher warfarin dose (3.03 ± 0.28 mg/day, 7.19 mg/day, respectively) than AA carriers (2.52 ± 0.07 mg/day; *P* < 0.001). Carriers of *CYP4F2* rs2108622 CT/TT genotypes required significantly higher warfarin dose (3.38 ± 0.22 mg/day, 2.79 ± 0.19 mg/day, respectively) than CC carriers (2.41 ± 0.08 mg/day; P < 0.001). However, the warfarin dose for carriers of *NQO1* rs1800566 CT/TT genotypes (2.46 ± 0.24 mg/day, 3.01 ± 0.27 mg/day, respectively) was not significantly higher than that for the CC carriers (2.33 ± 0.1 mg/day). The multiple linear regression model including genotypes and demographic characteristics, could explain 20.1% of individual variations in the daily stable warfarin dose in Sichuan Han Chinese. *VKORC1* rs9923231 contributed most (15%) to the individual variations in daily stable warfarin dose, while *CYP4F2* rs2108622 contributed least (3%).

**Conclusion:**

*NQO1* rs1800566 is not a significant genetic factor of warfarin dose for Han Chinese, whereas *VKORC1* rs9923231 and *CYP4F2* rs2108622 are significant genetic factors, which could explain 15% and approximately 3% of individual variations in the daily stable warfarin dose respectively.

## Background

Atrial fibrillation (AF) is one of the most common cardiac rhythm disorders [1]. The population of AF cases aged ≥35 years in China is 5.26 million according to 2010 Chinese Census and the number of AF ablation is increasing rapidly. Catheter ablation is superior to antiarrhythmic drugs in maintaining sinus rhythm [1]. The perioperative stage of atrial fibrillation catheter ablation refers to 3 weeks before operation, intraoperative period and 3 months after surgery, which requires anticoagulant therapy to cooperate with surgical treatment. Catheter ablation is a complex procedure with high risk of asymptomatic stroke during this period, hence, the management of patients should be treated with discretion. There is highly consistent evidence from observational studies that a continuous warfarin strategy during radiofrequency catheter ablation of AF reduces the risk of thromboembolic complications without increasing the risk of bleeding. Warfarin, an oral anticoagulant drug, is prescribed for patients who have undergone a catheter ablation procedure for treating atrial fibrillation. However, because of ethnic and individual dosing variations, warfarin dosage should be based on the International Normalized Ratio (INR) and carefully regulated [2]. Inadequate warfarin dosage could lead to harmful effects such as bleeding and thromboembolism, especially at the initial treatment phase [3].

Warfarin dosage has been found to be significantly associated with single nucleotide polymorphisms (SNPs) in the genes, vitamin K epoxide reductase complex subunit 1 (*VKORC1*) and cytochrome P450 complex subunit 4F2 (*CYP4F2*) [4–12]. However, SNPs in *VKORC1* and *CYP4F2* are reported to be significantly different due to different ethnic backgrounds [5, 11, 13–16]. The G allele of *VKORC1*–1639 and the C allele of *VKORC1*–1173, which are responsible for higher stable warfarin dose, were present in higher frequencies in Caucasian than in the Han Chinese population [14–17]. The T allele of *CYP4F2* rs2108622, also responsible for higher stable warfarin dose [5], had a higher frequency in Indian and Caucasian population than in the Chinese and African-American population [11].

The dicoumarol-sensitive NAD(P)H: quinone oxidoreductase 1 (NQO1) catalyzes the two-electron reduction of several quinones, including vitamin K (K1, K2 and K3). NQO1 is thought to reduce vitamin K to vitamin K hydroquinone that functions as a co-factor for the γ-carboxylation and further activation of clotting factors [18]. Thus, the *NQO1* gene is considered to be an additional candidate genetic factor that can affect warfarin dose. However, this hypothesis is challenged by two findings. Firstly, NQO1-deficient mice dosed with warfarin are able to reduce vitamin K and do not manifest a bleeding problem, questioning the role of NQO1 in vitamin K reduction [19, 20]. Secondly, SNPs in the *NQO1* gene exhibit ethnic variation. The T allele of *NQO1* rs1800566, which is responsible for a higher stable warfarin dose [4], has higher frequency in Hispanic-Americans than in African-Americans [4]. Besides, NQO1 is found to have a significant association with warfarin dose in Hispanic-Americans [4], but is not associated with warfarin dose in African-Americans [21]. Till date, no studies have been performed in the Sichuan Han Chinese population. Therefore, it is important to determine if *NQO1* variants also influence warfarin dose in the Han Chinese patients.

Herein, we examined the impact of ethnic variation in *VKORC1*, *CYP4F2* and *NQO1* genes on warfarin dosage in the Han Chinese patients.

## Methods

### Study subjects

Patients enrolled in this study must meet five requirements: (1) underwent a catheter ablation procedure for atrial fibrillation; (2) received oral warfarin at a stable dose for at least 3 weeks prior to catheter ablation and 3 months after ablation; (3) their INR values controlled in a range of 1.5 to 3.0; (4) Han Chinese living in the Sichuan Province; (5) did not have a history of liver dysfunction or liver enzymes > 3 times the upper limit of normal. A total of 222 patients (82 males and 140 females) in West China Hospital were studied. Their demographic information including age, body weight, height and stable warfarin dosage was carefully recorded, and their blood samples were collected for genotype determination. The study population included had high (≥2) risk according to the CHA2DS2-VASc risk score. The Ethics Committee of West China Hospital, Sichuan University approved this study. All participants provided their written informed consent to participate in this study. The ethics committees approved this consent procedure.

### Genotyping of polymorphisms

Genomic DNA was isolated from blood samples using a commercially available kit (QIAamp DNA Blood Mini Kit, Qiagen, CA). SNPs, including *VKORC1* rs9923231, *CYP4F2* rs2108622 and *NQO1* rs1800566, were determined by a polymerase chain reaction-restriction fragment length polymorphism (PCR-RFLP) method. All PCR reactions were carried out in a 25 μl volume containing 50 ng genomic DNA, 2.5 mM dNTPs, 10 mM each of forward and reverse primers, 2.5 ml 10X Ex Taq buffer and 0.75 U Ex Taq DNA polymerase (Takara, Japan). PCR thermocycling consisted of an initial denaturation period of 5 min at 95 °C; then 40 cycles of a denaturation at 95 °C for 30 s, an annealing period of 30 s at 62 °C for *NQO1*, 58 °C for *CYP4F2*, 61 °C for *VKORC1*, an extension period for 45 s at 72 °C; and a final extension for 10 min at 72 °C. Details on forward/reverse primer sequences, amplified product sizes and restriction enzymes used are shown in Table 1. Genotyping results were confirmed by repeating the reactions twice.

**Table 1:** Single Nucleotide Polymorphism Genotyping based on the PCR-RFLP method

SNP	Forward (F) and Reverse (R) Primer Sequence (5' → 3')	Extended size(bp)	Restriction Enzyme	Reference
NQO1 rs1800566	F: AAGCCCAGACCAACTTCT R: GCGTTTCTTCCATCCTTC	196	Hinf I	[3, 16]
VKORC1 rs9923231	F: ATCCCTCTGGGAAGTCAAGC R: CACCTTCAACCTCTCCATCC	636	BcnI	[4]
CYP4F2 V433 M rs2108622	F: AGTCCCGGTCATCTCCCGCCAT R: CGCCAGCCTTGGAGAGACAGACA	358	PvuII	[4]

*SNP* single nucleotide polymorphism. *bp* base pair

### Statistical analysis

The frequency distributions of *VKORC1* rs9923231, *CYP4F2* rs2108622 and *NQO1* rs1800566 were tested for deviation from Hardy-Weinberg equilibrium using the χ2-test. The differences in warfarin dose between the groups categorized by SNPs were analyzed by a two-sample *t*-test. Variables (age, body surface area, gender and variation of SNPs) associated with warfarin dose were analyzed by Pearson’s correlation coefficient and the χ2-test. Variables with *P* ≤ 0.1, and not strongly correlated with other factors, were analyzed by multiple linear regression analysis to find their joint association with warfarin dose. Data were analyzed using SPSS ver.17.0 (SPSS Inc., Chicago, IL). *P* < 0.05 was considered to be statistically significant. Data are shown as number (percentage) or mean ± SD (range).

## Results

### Patient demographic and clinical characteristics

A total of 222 atrial fibrillation patients taking stable warfarin therapy before catheter ablation procedure were studied, and their demographic and clinical characteristics are summarized in Table 2. Patient age ranged from 21 to 71 years, with a mean age of 47.5 ± 0.72 years. Body surface area of patients ranged from 1.3 to 2.35 m^2^, with a mean of 1.7 ± 0.01 m^2^. 222 patients had successfully controlled their INR values to a range of 1.5 to 3.0 with a daily stable warfarin dose (2.05 ± 0.05 mg/day).

**Table 2:** Demographic and clinical characteristics of Sichuan Han Chinese patients with catheter ablation of atrial fibrillation

Variable	Patients(*n* = 222)
Mean Age ± SD(Range)(years)	47.5 ± 0.72(21–71)
Male	47.1 ± 1.23(21–71)
Female	47.7 ± 0.899(23–67)
Gender	
Male	82(36.9%)
Female	140(63.1%)
Mean Body Surface Area ± SD (Range) (m^2^)	1.7 ± 0.01(1.3–2.35)
Male	1.84 ± 0.02(1.53–2.35)
Female	1.63 ± 0.01(1.3–2.07)
Stable warfarin dose ± SD (mg /day)	2.05 ± 0.05
Average INR	
1.5–2.0	120(54.1%)
2.0–2.5	83(37.4%)
2.5–3.0	19(8.5%)

The data are shown as mean ± SD. Range: the data range from the lowest value to the highest. %: percentage. INR: International Normalized Ratio

### Genotype analysis

Genotypes of *VKORC1* rs9923231, *CYP4F2* rs2108622 and *NQO1* rs1800566 were successfully determined for 222, 221 and 116 patients, respectively. The genotyping results in Table 3 showed that the frequency of the mutated allele of *CYP4F2* rs2108622 and *NQO1* rs1800566 was low - 19.5% of all patients carried the T allele of *CYP4F2* rs2108622 and 13% carried the T allele of *NQO1* rs1800566. In contrast, the frequency of the mutated allele of *VKORC1* rs9923231 was high - 90% patients carried the A allele of *VKORC1* rs9923231, while only 1 patient carried the wild type (GG). The genotype distributions of *VKORC1* rs9923231 and *NQO1* rs1800566 are in Hardy-Weinberg equilibrium,except *CYP4F2* rs2108622.

**Table 3:** The allelic frequencies and genotype distributions in Sichuan Han Chinese patients with catheter ablation of atrial fibrillation

Gene	SNP	Genotype	Patient Number (%)	Mean stable warfarin dose±SD (mg/day)	Allele	Frequency Number (%)
NQO1	rs1800566	CC	90(78)	2.33 ± 0.1	C	202(87)
		CT	22(19)	2.46 ± 0.24	T	30(13)
		TT	4(3)	3.01 ± 0.27		
VKORC1	rs9923231	GG	1(0.4)	7.19	G	45(10)
		AG	43(19)	3.03 ± 0.28	A	399(90)
		AA	178(80)	2.52 ± 0.07		
CYP4F2 V433 M	rs2108622	CC	155(70)	2.41 ± 0.08	C	356(80.5)
		CT	46(20)	3.38 ± 0.22	T	86(19.5)
		TT	20(9)	2.79 ± 0.19		

*SNP* single nucleotide polymorphism

### Impact of *VKORC1* rs9923231, *CYP4F2* rs2108622 and *NQO1* rs1800566 SNPs on the daily stable warfarin dose

**Fig. 1:**
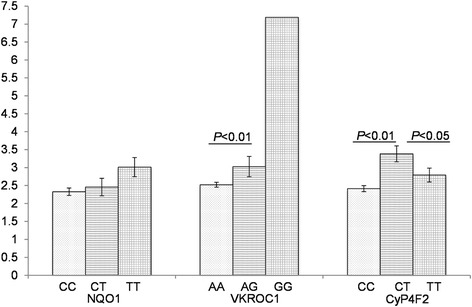
Mean stable warfarin dose ± SD (mg/day) by *VKORC1* rs9923231, *CYP4F2* rs2108622 and *NQO1* rs1800566 genotypes. The data of Mean stable warfarin dose ± SD are from Table 3. The differences in warfarin dose between the groups categorized by SNPs were analyzed by a two-sample *t*-test. *P* < 0.05 was considered to be statistically significant

The association of the daily stable warfarin dose with *VKORC1* rs9923231, *CYP4F2* rs2108622 and *NQO1* rs1800566 genotypes is shown in Table 3 and Fig. 1. With regard to the *VKORC1* rs9923231 genotype, the AG heterozygote carriers required a significantly higher warfarin dose (3.03 ± 0.28 mg/day; *P* < 0.001) than the AA carriers (2.52 ± 0.07 mg/day), while the single carrier of wild type GG required a much higher dose (7.19 mg/day) than the AG and AA carriers. In patients with *CYP4F2* rs2108622 genotype, the CT and TT carriers required a significantly higher warfarin dose (3.38 ± 0.22 mg/day, *P* < 0.001; 2.79 ± 0.19 mg/day, *P* < 0.001, respectively) than the CC wild type carriers (2.41 ± 0.08 mg/day). With regard to the *NQO1* rs1800566 genotype, the warfarin dose for the CT and TT carriers (2.46 ± 0.24 mg/day and 3.01 ± 0.27 mg/day, respectively) was not significantly higher than the CC wild type carriers (2.33 ± 0.1 mg/day).

### Contribution of demographic characteristics and genotype variation to the daily stable warfarin dose

Multiple linear regression analysis was used to investigate the contribution of demographic characteristics and genotype variation to the daily stable warfarin dose in Sichuan Han Chinese population. Our data indicate that several factors (*VKORC1* rs9923231, *CYP4F2* rs2108622, *NQO1* rs1800566, age, weight, height and gender) contributed to 20.1% of individual variations in the daily stable warfarin dose (Table 4). *VKORC1* rs9923231 exhibited a significant correlation with the daily stable warfarin dose (*P* < 0.001) and contributed the most (15%) to individual variations in the daily stable warfarin dose. *CYP4F2* rs2108622 also contributed approximately 3% to variation in the daily stable warfarin dose (*P* < 0.001). However, *NQO1* rs1800566 was not significantly associated with the daily stable warfarin dose (*P* = 0.12).

**Table 4:** Demographic and genetic factors associated with daily stable warfarin dose by multiple linear regression

Factors	Standardized coefficient	*P*-value
Age	−0.077	< 0.001
Gender	−0.14	< 0.001
Body Surface Area	−0.05	< 0.001
SNP of *VKORC1*	0.150047	< 0.001
SNP of *CYP4F2*	−0.0299	< 0.001
SNP of *NQO1*	0.087033	0.12

Variables (age, body surface area, gender and variation of SNPs) associated with warfarin dose were analyzed by Pearson’s correlation coefficient and the χ2-test. Variables with *P* ≤ 0.1, and not strongly correlated with other factors, were analyzed by multiple linear regression analysis to find their joint association with warfarin dose. *P* < 0.05 was considered to be statistically significant

## Discussion

In this study, the effect of *VKORC1* rs9923231, *CYP4F2* rs2108622 and *NQO1* rs1800566 genotypes on the daily stable warfarin dose in Sichuan Han Chinese patients with catheter ablation of atrial fibrillation was determined, and the allelic frequency and genetic distribution of these genotypes were also compared with other ethnic populations. To our knowledge, this is the first study that investigates the effect of the *NQO1* rs1800566 genotype on warfarin dose in Han Chinese patients. Till date, such studies have been performed in African-American and Hispanic-American patients [4, 21], but not in Han Chinese patients. Given the ethnic variation of *NQO1,* there was a critical need for assessing its impact on the daily stable warfarin dose in Han Chinese patients in this study.

The allele*s* identified to be responsible for higher warfarin dose requirement, *VKORC1* rs9923231, *CYP4F2* rs2108622 and *NQO1* rs1800566, were present at a low frequency in Sichuan Han Chinese patients, which might explain their sensitivity to warfarin and their requirement for a lower warfarin dose as compared to Caucasians. The allelic frequencies and genetic distributions of *VKORC1* rs9923231 in Han Chinese patients were different from Caucasian patients, the G allele of *VKORC1* rs9923231 had lower frequency in Han Chinese patients than in Caucasian patients. This finding might provide a reason to the different warfarin dose requirement between these two populations [14, 16]. This finding also suggests that *VKORC1* is the genetic factor for ethnic variations in warfarin dose. However, the allelic frequencies and genetic distributions of the *CYP4F2* rs2108622 in Han Chinese patients were not different from Caucasian patients, confirming a previous study that suggested that *CYP4F2* did not influence ethnic variations in warfarin dose [5]. The allelic frequency and genetic distribution of *NQO1* rs1800566 in Han Chinese patients were not different from the African-American population either [21], suggesting that *NQO1* is also not a genetic factor in modulating ethnic variation in warfarin dose.

Multiple linear regression analysis demonstrated that *VKORC1* rs9923231 and *CYP4F2* rs2108622 associated significantly with the daily stable warfarin dose (*P* < 0.001) and contributed 15 and 3% to individual variation in the daily stable warfarin dose, respectively. These results suggests that *VKORC1* rs9923231 and *CYP4F2* rs2108622 are significant genetic factors contributing to individual differences in warfarin dose within an ethnic population, as also indicated in previous studies [5, 8, 10, 11, 13, 22, 23]. Therefore, monitoring of these two genotypes could help determine the personalized warfarin dose requirement in Han Chinese patients. Consistent with a previous study in African-American patients [21], *NQO1* rs1800566 is not significantly associated with the daily stable warfarin dose in Han Chinese patients as well, suggesting that *NQO1* does not contribute to individual variations in warfarin dose within an ethnic population. Together with an in vitro study demonstrating that *VKORC1* plays a major role in vitamin K cycle whereas other enzymes play smaller roles [24], our study suggests that *NQO1* may not be a significant genetic factor influencing warfarin dose.

While our data are limited by our small sample size, the sample we collected were almost Han Chinese and the other nationalities will be researched in the future.

## Conclusion

Our current data support *VKORC1* and *CYP4F2*, but not NQO1, genotypes in prediction of warfarin dose requirements in Han Chinese. Moving forward, developing the predictive ability that take genetic characteristics into account may help improve dose prescribing and reduce the risk of bleeding and thrombosis during the warfarin initiation period. Formal algorithm establishment will require a large sample.

## Abbreviations

AF: atrial fibrillation
bp: base pair
INR: International Normalized Ratio
PCR-RFLP: polymerase chain reaction restriction fragment length polymorphism
SNP: single nucleotide polymorphisms
